# Assessment of treatment outcomes: cytoreductive surgery compared to radiotherapy in oligometastatic prostate cancer – an in-depth quantitative evaluation and retrospective cohort analysis

**DOI:** 10.1097/JS9.0000000000001308

**Published:** 2024-03-18

**Authors:** Bisheng Cheng, Haixia He, Bingliang Chen, Qianghua Zhou, Tianlong Luo, Kaiwen Li, Tao Du, Hai Huang

**Affiliations:** a Department of Urology; b Department of Obstetrics and Gynecology; c Guangdong Provincial Key Laboratory of Malignant Tumor Epigenetics and Gene Regulation; d Department of Radiation Oncology; e Guangdong Provincial Clinical Research Center for Urological Diseases, Sun Yat-Sen Memorial Hospital, Sun Yat-Sen University; f Department of Urology, Sun Yat-sen University Cancer Center, Guangzhou; g Department of Urology, The Sixth Affiliated Hospital of Guangzhou Medical University, Qingyuan People’s Hospital, Qingyuan, Guangdong; h Department of Urology, Fujian Medical University Union Hospital, Fuzhou, People’s Republic of China

**Keywords:** cytoreductive surgery, oligometastatic, prostate cancer, radiotherapy, retrospective study

## Abstract

**Background:**

The management of oligometastatic prostate cancer, defined by its few metastatic sites, poses distinct clinical dilemmas. Debates persist regarding the most effective treatment approach, with both cytoreductive surgery and radiotherapy being key contenders. The purpose of this research is to thoroughly evaluate and compare the effectiveness of these two treatments in managing patients with oligometastatic prostate cancer.

**Methods:**

A comprehensive search of the literature was carried out to find pertinent publications that compared the results of radiation and cytoreductive surgery for oligometastatic prostate cancer. A meta-analysis was conducted in order to evaluate both short-term and long-term survival. Furthermore, utilizing institutional patient data, a retrospective cohort research was conducted to offer practical insights into the relative performances of the two treatment regimens.

**Results:**

Five relevant studies’ worth of data were included for this meta-analysis, which included 1425 patients with oligometastatic prostate cancer. The outcomes showed that, in comparison to radiation, cytoreductive surgery was linked to a substantially better cancer-specific survival (CSS) [hazard ratio (HR): 0.70, 95% (CI): 0.59–0.81, *P*<0.001] and overall survival (OS) [HR, 0.80; 95% (CI), 0.77–0.82; *P*<0.01]. The two therapy groups’ Progression-Free Survival (PFS) and Castration-Resistant Prostate Cancer-Free Survival (CRPCFS), however, did not differ significantly (HR: 0.56, 95% CI: 0.17–1.06; HR: 0.67, 95% CI: 0.26–1.02, respectively). Out of the 102 patients who were recruited in the retrospective cohort research, 36 had cytoreductive surgery (CRP), 36 had radiation therapy (primary lesion), and 30 had radiation therapy (metastatic lesion). The follow-up time was 46.3 months (18.6–60.0) on average. The enhanced OS in the CRP group [OS interquartile range (IQR): 45–60 months] in comparison to the radiation group (OS IQR: 39.0–59.0 months and 25.8–55.0 months, respectively) was further supported by the cohort research. Furthermore, CRP had a better OS than both radiation (primary region) and radiotherapy (metastatic region), with the latter two therapeutic methods having similar OS.

**Conclusion:**

This meta-analysis and retrospective research provide valuable insights into the comparative efficacy of cytoreductive surgery and radiotherapy for oligometastatic prostate cancer. While short-term survival (PFS, CRPCFS) was similar between the two groups, cytoreductive surgery exhibited superior CSS and OS. Adverse event rates were manageable in both modalities. These findings contribute to informed treatment decision-making for clinicians managing oligometastatic prostate cancer patients. Further prospective studies and randomized controlled trials are essential to corroborate these results and guide personalized therapeutic approaches for this distinct subset of patients.

## Introduction

Highlights

- A comprehensive review and meta-analysis to compare cytoreductive surgery versus radiotherapy in oligometastatic prostate cancer treatment.
- Inclusion of data from five studies encompassing 1425 patients for robust comparative efficacy evaluation.
- Cytoreductive surgery associated with significantly improved overall survival compared to radiotherapy.
- No significant difference in progression-free survival between the two treatment modalities.
- Retrospective cohort study supports the superior overall survival with cytoreductive surgery.
- Findings aid in informed decision-making and underscore the need for further studies to refine treatment approaches.

Oligometastatic prostate cancer represents a distinct clinical entity within the spectrum of metastatic disease, characterized by a limited number of metastases predominantly localized to specific anatomical sites^1^. This intermediate stage between localized and widespread metastases has sparked considerable interest in exploring optimal treatment strategies that may prolong survival and enhance the quality of life for affected patients^2–4^. Among the therapeutic modalities gaining prominence are cytoreductive surgery and radiotherapy^5,6^, each offering distinct advantages and potential outcomes.

Cytoreductive surgery involves the surgical removal of metastatic lesions with the intent of reducing tumor burden and potentially delaying disease progression^7–9^. Radiotherapy, on the other hand, delivers targeted ionizing radiation to cancer cells, aiming to achieve local tumor control and alleviate symptoms^10–12^. Both approaches have demonstrated efficacy in certain clinical settings, but a comprehensive comparison of their relative benefits and limitations is essential to guide clinical decision-making. While clinical trials have contributed to our understanding of these treatments, they often have limitations in terms of patient selection and follow-up duration. In addition, real-world retrospective cohort studies, by virtue of their inclusion of broader patient populations and longer-term outcomes, can provide valuable insights into the practical application and outcomes of cytoreductive surgery and radiotherapy in the management of oligometastatic prostate cancer.

In this context, we conducted a comprehensive meta-analysis and retrospective cohort study to systematically compare the efficacy of cytoreductive surgery with radiotherapy in treating oligometastatic prostate cancer. By synthesizing existing evidence from the literature and evaluating the outcomes of a well-defined cohort of patients, we sought to address critical questions regarding overall survival, progression-free survival, and treatment-related adverse events associated with these interventions.

This study contributes to the growing body of knowledge surrounding the management of oligometastatic prostate cancer, shedding light on the practical effectiveness and potential benefits of cytoreductive surgery and radiotherapy. The insights gained from this research are poised to inform clinical decision-making and guide the development of personalized treatment strategies for patients at this intermediate stage of disease progression. Furthermore, the findings may underscore the importance of considering real-world evidence in shaping treatment guidelines and optimizing patient outcomes in the context of oligometastatic prostate cancer.

## Methods

The results of this investigation are presented in compliance with academic paper standards by following the recommendations of PRISMA (Preferred Reporting Items for Systematic Reviews and Meta-Analyses)^13^, STROCSS (Strengthening the reporting of cohort, cross-sectional and case–control studies in surgery)^14^ 2021 criteria and AMSTAR (Assessing the methodological quality of systematic reviews)^15^. The following page, https://www.crd.york.ac.uk/prospero, has more comprehensive information regarding the registration procedure.

### Study design

To find pertinent studies reporting CRP findings in OmPCa patients up to October 2023, a thorough search of the main medical databases, including PubMed, Embase, Web of Science, and CNKI, was conducted. Appropriate keywords for “oligometastatic prostate cancer,” “cytoreductive surgery,” and “rediotherapy” were included in the search approach.

### Meta-analysis

#### Search of literature

A comprehensive exploration of prominent medical databases, such as PubMed, Embase, Web of Science, and CNKI, was carried out to find pertinent research articles detailing the results of cytoreductive surgery for patients with oligometastatic prostate cancer till October 2023.

#### Inclusion and exclusion criteria

The literature search was carried out by two impartial reviewers, and papers were included in the meta-analysis provided they satisfied the following requirements:

1. Study design and participants: Included were studies that discussed the results of CRP in OmPCa patients. Histopathological testing revealed the presence of PCa in all cases. Imaging modalities such as CT (computed tomography), MRI (magnetic resonance imaging), or ECT (emission computed tomography) were used to assess the tumor metastatic status of the patients.
2. Intervention measures: Radical and transurethral prostatectomy were among the tumor reduction treatments that the experimental group underwent. Radiotherapy was accepted by the control group.
3. Prognostic survival analysis in individuals with oligometastatic prostate cancer needs to be the primary focus of the study. In order to enable survival analysis, studies should include enough follow-up data, guaranteeing that the survival rate at every follow-up time point may be obtained either directly or indirectly from the study’s original text.
4. Study type: The meta-analysis deemed both retrospective studies and randomized controlled trials (RCTs) suitable for incorporation.

Exclusion criteria:

1. Study types: The meta-analysis excluded review articles, secondary literature, case reports, conference abstracts, and other types of non-primary research.
2. Animal research: Studies involving animal research were not considered for inclusion in the meta-analysis.
3. Oligometastases definition: Studies that focused on transitional states not meeting the defined criteria for oligometastases were excluded from the meta-analysis.
4. Unavailability of full text: Studies for which the full text could not be obtained through various methods were excluded.
5. Insufficient data: Studies lacking relevant outcome data or having incomplete information essential for the meta-analysis were excluded.

#### Data extraction and quality assessment

Literature screening was conducted independently by two researchers according to the above criteria. And then, the data were systematically compared. In cases of discordance, the researchers engage in discussion to reconcile their opinions. If a consensus still remains elusive, professionals in the field are consulted for further input. The following data components were retrieved from the publications after a thorough investigation was conducted: first author, publication year, median age, country, literature type, follow-up period, total patient count, intervention methods, survival analysis, HR, 95% CI, Gleason score, and TNM stage. Direct data is the main source used to extract the risk ratio and 95% CI, while data derived using the Kaplan–Meier approach is the backup option. Survival rates were calculated using Kaplan–Meier curves, and the HR index was calculated using techniques from Tierney *et al*.^16^. The quality of the studies was assessed using appropriate tools. The proper instruments were used to evaluate the studies’ quality. The Newcastle–Ottawa Scale (NOS) was used for non-randomized research, while the Cochrane Risk of Bias Tool^17^ was used for randomized controlled trials (RCTs). These instruments aided in the assessment of methodological quality as well as bias risk. Three areas are covered by the NOS literature quality assessment, which has eight sub-evaluation questions. On a scale of 0–9, the literature is given a score; a score of 6 or above is seen as indicative of good quality.

#### Statistical analysis

Meta-analyses were conducted to determine the HRs with relevant 95% CI based on a proper statistical approach. In cases of significant heterogeneity, random-effects models were applied to identify potential variations in different studies. Subgroup analyses were carried out to analyze the sources of heterogeneity, considering factors like study design, sample size, and treatment modality. Heterogeneity was determined based on the *I*^2^ tests. A *P*<0.05 for the Cochran *Q* statistic or an *I*^2^ statistic exceeding 50% indicated statistically significant heterogeneity among the studies. The random-effects model was applied under the condition of heterogeneity existed.

### Retrospective research analysis

#### Patient selection

In our retrospective analysis, we examined a cohort of patients diagnosed with oligometastatic prostate cancer who underwent treatment at our institution from January 2008 to August 2018. This cohort included individuals who received either cytoreductive surgery or radiotherapy (targeting either primary lesions or metastatic lesions). The selection of treatment modality was based on a multidisciplinary team’s decision, taking into account various factors such as the patient’s overall health, tumor characteristics, and patient preference. To mitigate selection bias, we also considered the influence of the physician’s strategy and any potential investigator preferences. These aspects were evaluated and controlled for in our analysis to ensure a balanced comparison between the two treatment groups.

#### Inclusion and exclusion criteria

The medical records of eligible patients were identified, and inclusion criteria for the retrospective analysis were as follows:

Primary diagnosis: Confirmed primary prostate cancer via histopathological analysis.

Limited metastatic spread: Evidence of up to five metastatic lesions, typically identified using imaging techniques such as CT, MRI, PET-CT, or bone scans.

Site of metastases: Common sites include bones, lymph nodes, and occasionally visceral organs.

Absence of widespread disease: No evidence of widespread metastatic disease or involvement of critical organs that would classify the disease as advanced or widespread metastatic prostate cancer.

Adequate follow-up data is available for survival analysis. Patients with incomplete data or those lost to follow-up were excluded from the analysis.

#### Data collection

Patient demographics (e.g. age, Gleason score), tumor characteristics (e.g. number and location of metastases), surgical outcomes (e.g. surgical complications), treatment-related events (e.g. radiotherapy-related adverse reactions), and survival rates (OS, PFS, CRPCFS) were collected from medical records and analyzed. The median follow-up time for the cohort was calculated.

#### Statistical analysis

Depending on the sample size and anticipated population frequencies, two tests were used to compare categorical variables: Pearson’s Chi-squared test and Fisher’s exact test. For continuous variables that did not match the normalcy criteria, the Mann–Whitney *U* test was used. Frequencies and percentages for categorical variables were used to display descriptive data. The means±standard deviation (SD) were used to characterize continuous parametric variables, while the median with the interquartile range was used to represent nonparametric variables. Using the Kaplan–Meier technique, survival rates were determined for castration-resistant prostate cancer-free survival (CRPCFS), progression-free survival (PFS), and overall survival (OS). The log-rank test was used to evaluate differences between groups. SPSS version 22.0 (IBM Corp., Armonk, New York, USA) and GraphPad Prism version 9.1.0 (GraphPad Software, San Diego, California, USA) were used for all statistical analyses. For all tests, statistical significance was defined as a two-sided *P* value of less than 0.05.

## Results

### Quantitative analysis

#### Study characteristics

**Figure 1:**
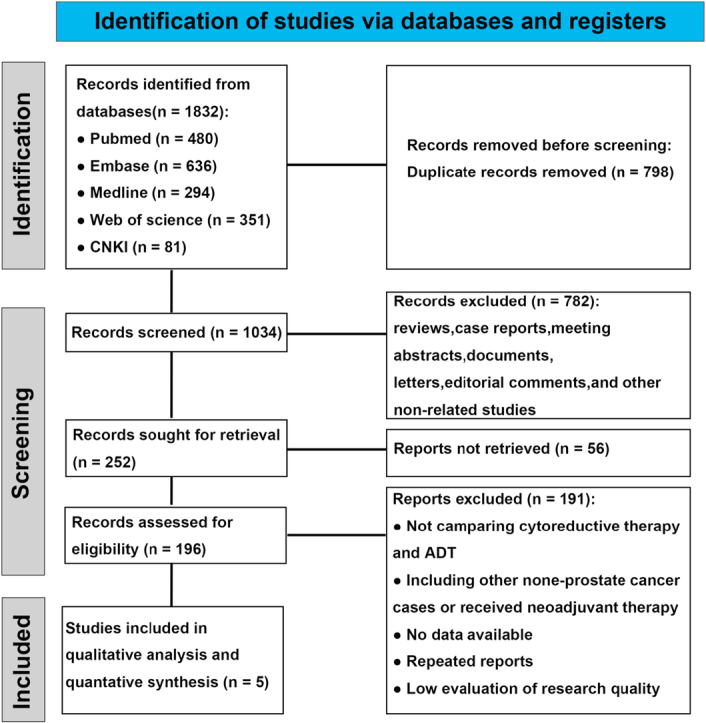
Flow diagram following the PRISMA template of the search strategy.

A total of 1832 relevant articles were initially screened. Among these, 798 duplicate articles were removed. Additionally, 782 articles, including basic experimental studies, case reports, and reviews, were excluded according to their titles and abstracts. According to the analysis results of the full-text version, 56 unreachable reports and 191 articles did not meet the inclusion criteria for the control group, lacked clear information on metastasis or had incomplete data for prognosis evaluation. Eventually, five studies^18–22^ were considered eligible for inclusion in the present study. These five studies encompassed a total of 1425 cases of oligometastatic prostate cancer (OmPCa), of which 808 cases were in the CRP group and 617 in the radiotherapy group. Details of the search process and methodology are provided in Figure 1, and the characteristics of the included studies are presented in Table 1. Among the selected studies, four were subjected to OS analysis, three to PFS analysis, four to CSS analysis, and two to CRPCFS analysis. Hazard ratios (HR) and their corresponding 95% confidence intervals (CI) were extracted either directly from the original articles or indirectly obtained from Kaplan–Meier curves using an EXCEL program developed by Tierney.

**Table 1:** Study characteristics for quantitative analysis.

References	Country	No. of patients	Age (median)	Interventions	T-stage	N-stage	M-stage (Lesions number)	Gleason score	PSA level (ng/ml)
Culp *et al*.^18^	USA	245/129	62/68	RP/Brachytherapy	T1/T2 130/91 T3/T4 113/20 Unknown 2/18	N0 165/89 N1 68/17 Unknown 12/23	M1a 17/8 M1b 14/8 M1c 71/38	NA	<10 115/45 10–19 50/19 20–29 17/11 ≥30 32/44 Unknown 31/10
Leyh *et al*.^20^	Germany	313/161	63/68	RP/RT	T≤cT2 280/137 T≥cT3 33/24	N0/Nx 288/147 N1 25/14	M1a 35/19 M1b 222/103 M1c 56/39	6 41/30 7 105/47 ≥ 8 145/72 Unknown 22/12	NA
Patel *et al*.^19^	USA	176/269	72/77	RP/RT	NA	NA	Unknown 1/1 Bone metastasis 82/146 Soft tissue metastasis 11/21 Soft tissue and bone metastasis 82/101	NA	Median 20.6/34.1
Xue *et al*.^21^	China	26/32	65.5/67.5	RP/SBRT	cT1-2 5/5 cT3a 13/12 cT3b-4 8/15	N0 20/21 N1 6/11	1–3 20/22 4–5 6/10	6 0/0 7 16/24 8 7/3 ≥ 9 3/5	Median 35.3/36.4
Lumen *et al*.^22^	Belgium	48/26	64/70	CRP/RT	T≤cT2 17/5 T≥cT3 31/21	N0 15/4 N1 33/22	M1a 23/9 M1b 25/17	NA	Median 19/40

CRP, cytoreductive radical prostatectom; IQR, interquartile distance; NA, no data available; RP, radical prostatectomy; RT, radiotherapy; SBRT, stereotactic body radiation therapy.

### Quality assessment

The quality assessment results, using either the Cochrane Risk of Bias tool or the Newcastle–Ottawa Scale (NOS), are presented in Tables 2 and 3. Regarding one randomized controlled trial (RCT)^22^, it revealed a risk of blinding (detection bias) due to its open-label nature. The non-RCT studies received scores ranging from six to seven points on the NOS, indicating relatively good methodological quality. In two specific studies^20,21^, there was a disparity in treatment modality between the two groups based on the clinical judgment of the practitioners. Except for concerns related to control selection and non-response rates, no other significant issues that could affect the quality of the studies were identified.

**Table 2:** Assessing the quality of randomized controlled trials using the Cochrane Risk of Bias Tool.

Study (country)	Random sequence generation (selection bias)	Allocation concealment (selection bias)	Blinding of participants and personnel (performance bias)	Blinding of outcome assessment (detection bias)	Incomplete outcome data addressed (attrition bias)	Selective reporting (reporting bias)	Other bias
Lumen (Belgium)	Low risk	Low risk	High risk	High risk	Low risk	Low risk	Unclear

**Table 3:** Quality assessment of non-randomized studies using the Newcastle–Ottawa Scale.

	Selection (4)				Comparability (2)	Exposure (3)				
Study (country)	Adequate definition of cases	Representativeness of cases	Selection of controls	Definition of controls	Control for important factor or additional factor	Ascertainment of exposure	Same method of ascertainment for cases and controls	Non-response rate	Total score	
Culp (USA)	1	1	0	1	2	1	1	0	7	
Leyh (Germany)	1	1	0	1	2	1	0	0	6	
Patel (USA)	1	1	0	1	2	1	1	0	7	
Xue (China)	1	1	0	1	2	1	0	0	6	

### Efficacy of cytoreductive surgery *versus* radiotherapy

#### Progression-free survival (PFS)

**Figure 2:**
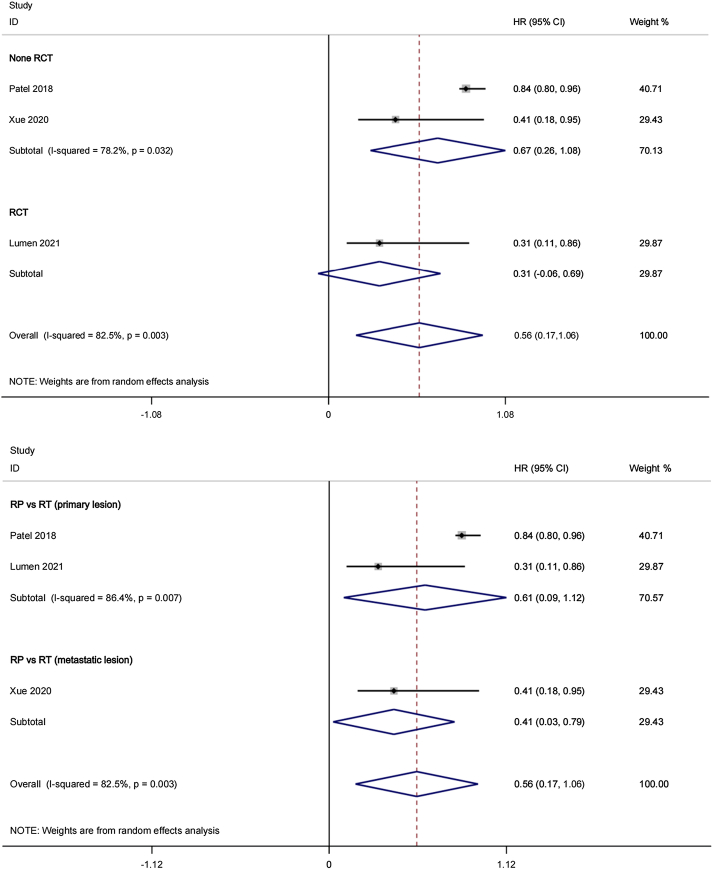
-1 Forest plot of progression-free survival in CRP group and RT group (Subgroup: RCT and Non-RCT). 2-2 Forest plot of progression-free survival in CRP group and RT group (Subgroup: RT (Primary lesion) and RT (Metastatic lesion)).

The comparison of progression-free survival (PFS) according to cytoreductive prostatectomy (CRP) and radiotherapy (RT) for oligometastatic prostate cancer (OmPCa) involved a total of three studies comprising 577 patients. Heterogeneity across the studies was also identified, with Cochran’s *Q* statistic showing a *P* value of 0.003 and an *I*^2^ statistic of 82.5%. In the meta-analysis encompassing all three studies, no significant difference in PFS was found (random effects HR: 0.56, 95% CI: 0.17–1.06, *P*>0.05). Figure 2-1, 2-2 presents the forest plots illustrating the PFS analysis for visual representation.

#### Castration-resistant prostate cancer-free survival (CRPCFS)

**Figure 3:**
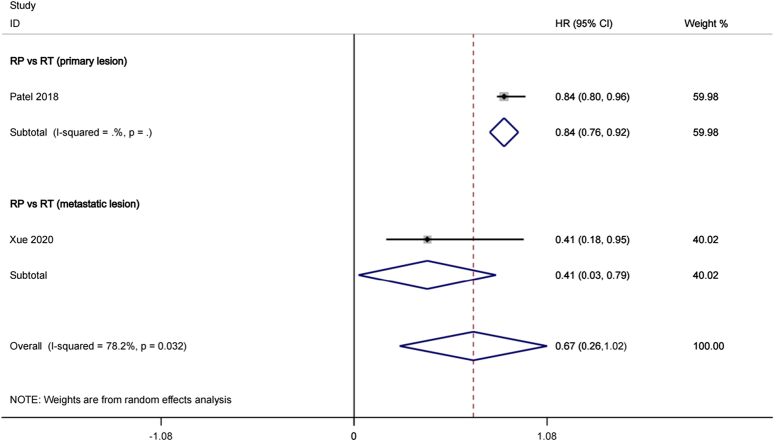
Forest plot of CRPC-free survival in CRP group and RT group.

A total of nine studies^20,21^ involving 503 patients were included in the comparison of time to castration-resistant prostate cancer (CRPC) in association with continuous androgen deprivation therapy (ADT) for oligometastatic prostate cancer (OmPCa). This analysis revealed certain heterogeneity across the studies, as evidenced by Cochran’s *Q* statistic (*P*=0.175) and *I*^2^ statistic (78.2%). No significant differences were observed in progression-free survival (random effects HR: 0.67, 95% CI: 0.26–1.02, *P*>0.05). Figure 3 presents forest plots of the CRPC-free survival analysis for visual representation.

#### Cancer-specific survival (CSS)

**Figure 4:**
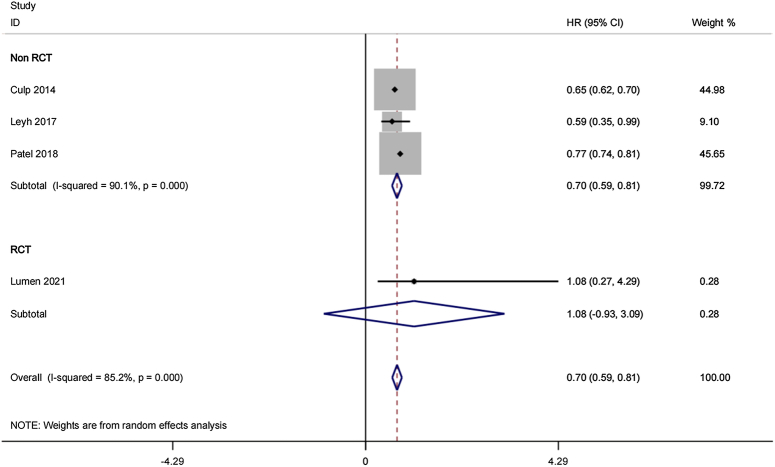
Forest plots of cancer-specific survival.

Up to four researches (with 1347 cases) were included in comparing CSS based on CRP and RT for OmPCa. According to the analysis result, there exist obvious differences [random effects HR, 0.70; 95% (CI), 0.59–0.81; *P*<0.01] reinforcing the notion that CRP leads to better survival outcomes in oligometastatic prostate cancer patients. Heterogeneity existed in different researches (*P*=0.0001; *I*^2^=85.2%) (Fig. 4).

#### Overall survival (OS)

**Figure 5:**
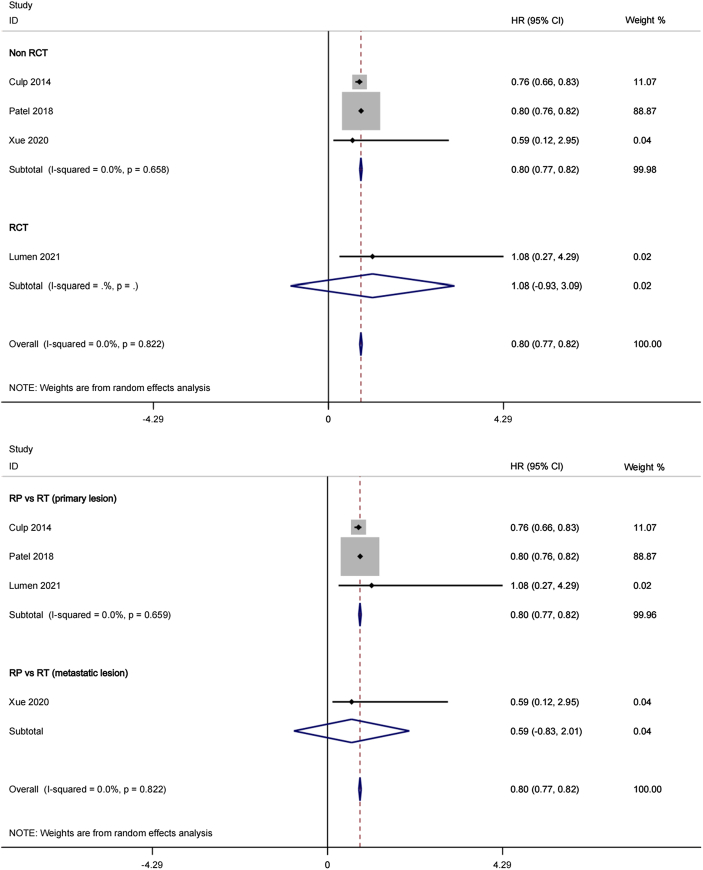
-1 Forest plots of overall survival (Subgroup: RCT and Non-RCT). 5-2 Forest plots of overall survival (Subgroup: RT (Primary lesion) and RT (Metastatic lesion)).

Four studies with 1091 cases were included in the comparison of OS between the two groups for OmPCa. Initial findings reflected that OS was obviously higher in the CRP group [HR, 0.80; 95% (CI), 0.77–0.82; *P*<0.01], with no notable heterogeneity identified across the studies (Cochran’s *Q* statistic, *P*=0.822). Forest plots depicting the OS data are provided in Figure 5-1, 5-2 for visual representation. The results of further subgroup analysis indicate that patients receiving primary radiotherapy demonstrate a higher overall survival rate compared to those undergoing radiotherapy solely for metastatic disease (Figure 5-2).

### Retrospective study

In this section, we present the findings of the retrospective research analysis that aimed to compare the outcomes of patients with oligometastatic prostate cancer who underwent cytoreductive surgery (CRS) versus those who received radiotherapy (RT) at our institution between January 2008 and August 2018.

A total of 102 patients with oligometastatic prostate cancer were included in the retrospective analysis. Among them, 36 patients underwent cytoreductive surgery (CRS group), and the remaining 66 patients received radiotherapy (either to the primary lesion or metastatic lesions). The two groups were well-matched in terms of baseline characteristics, ensuring a fair comparison. The median follow-up time for the entire cohort was 46.3 months, with a range between 18.6 and 60.0 months (Tables 4, 5). It is important to note that all patients in this study, regardless of whether they underwent CRS or RT, received endocrine supplemental therapy as part of their post-treatment regimen. This inclusion of endocrine therapy across both treatment groups was integral to our analysis, allowing for a more uniform comparison of outcomes between the CRS and RT cohorts. The impact of this consistent approach to hormonal therapy on patient outcomes was carefully considered and controlled for in our analysis, ensuring that our comparative evaluation of cytoreductive surgery and radiotherapy reflects a comprehensive treatment perspective.

**Table 4:** Baseline characteristics of included oligometastatic prostate cancer patients.

Characteristics	CRP (*n*=36)	RT (Primary lesion) (*n*=36)	RT (Metastatic lesion) (*n*=30)	*P* ^b^	*P* ^c^	*P* ^d^
Age, median (IQR)	70.0 (66.0–74.8)	68.5 (65.3–74.0)	69.0 (64.0–71.3)	0.5203	0.2877	0.6190
PSA, ng/ml (median, IQR)^a^	19.0 (11.7–39.0)	16.8 (11.3–33.8)	19.3 (10.1–31.2)	0.7689	0.7035	0.7225
Biopsy GS, *n*				0.0595		
≤7	11	21	13			
8–10	27	15	17			
cT-stage, *n*				0.3097		
T1–2	16	19	19			
T3–4	20	17	11			
cN-stage, *n*				0.8507		
N0	28	26	22			
N1	8	10	8			
cM-stage, *n*				0.8016		
M1a	16	14	14			
M1b	20	22	16			
Number of distant lesions, *n*				0.6756		
1	6	12	8			
2	8	7	7			
3	7	6	3			
4	9	5	9			
5	6	6	3			

CRP, cytoreductive radical prostatectomy; GS, Gleason score; IQR, interquartile distance; PSA, prostate-specific antigen; RP, radical prostatectomy; RT, radiotherapy.

a At the time of diagnosis.

b Comparing RP with RT (Primary lesion) patients.

c Comparing RP with RT (Metastatic lesion) patients.

d Comparing RT (Primary lesion) with the RT (Metastatic lesion) cohort.

**Table 5:** Clinicopathological characteristics of 36 patients undergoing cytoreductive radical prostatectomy for prostate conditions.

Characteristics	CRP (*n*=36)
PSA, ng/ml (median, IQR)^a^	19.0 (11.7, 39.0)
Treated by initial ADT, *n* (%)	
No	14 (38.9)
Yes	22 (61.1)
Open surgery approach, *n* (%)	28 (77.8)
Pathological T stage, *n* (%)	
1–2	12 (33.3)
3–4	24 (66.7)
Pathological N stage, *n* (%)	
N0	16 (44.4)
N1	20 (55.6)
Positive surgical margins, *n* (%)	10 (27.8)
Postoperative Gleason score, *n* (%)	
≤7	11 (30.6)
8–10	25 (69.4)
PSA at 6 weeks after RP (ng/ml), *n* (%)	
<0.1	21 (58.3)
≥0.1 and <0.2	8 (22.2)
≥0.2	7 (19.4)

ADT, androgen deprivation therapy; CRP, cytoreductive radical prostatectomy; IQR, interquartile range; PSA, prostate-specific antigen.

a At the time of diagnosis.

During the perioperative period, a total of 10 cases (27.7%) of complications were observed within 90 days after cytoreductive surgery (CRP). Of these, four cases were classified as Clavien–Dindo grade 1, two cases as grade 2, and two cases as grade 3a. Two patients experienced more severe complications classified as grade 3b or 4a, namely a pelvic hematoma and an anastomotic stenosis, respectively. There were no grade 4b complications or perioperative deaths recorded. The most prevalent complication was urinary incontinence, accounting for 11.1% of cases accepted CRP after the first year, which decreased to 5.5% in the second year (Table 6).

**Table 6:** Complications in perioperative and postoperative phases of cytoreductive radical prostatectomy.

	Patients, *n* (%)
Perioperative complications^a^	
I	4 (11.1)
II	2 (5.5)
IIIa	2 (5.5)
IIIb	1 (2.7)
IVa	1 (2.7)
IVb	0
V	0
Total	10 (27.7)
Late postoperative (>90 days) symptomatic local events	
Urinary incontinence^b^	
At 1st year	4 (11.1)
At 2nd year	2 (5.5)
Urethral stricture	
Caused by hypertrophic scar	2 (5.5)
Caused by tumor recurrence at anastomotic stoma	1 (2.7)
Urethrorectal leakage caused by rectal injury at RP	0

a The grade of perioperative complications was evaluated according to Clavien–Dindo grade I–V complications.

b Urinary incontinence was defined as one or more pads per day.

We further evaluated radiotherapy-related events in patients undergoing radiotherapy using the Common Terminology Criteria for Adverse Events (CTCAE) scale and the Radiation Therapy Oncology Group (RTOG) scale. A total of seven patients experienced Grade 1 adverse reactions, according to CTCAE, while two patients experienced Grade 2 adverse reactions. No patients had adverse reactions of Grade 3 or above, according to CTCAE. On the other hand, nine patients experienced Grade 1 adverse reactions according to RTOG, and no other more severe adverse reactions were observed. The incidence of radiotherapy-related events showed no statistically significant difference between the RT (Primary lesion) group and the RT (Metastatic lesion) group. In conclusion, the above results suggest that radiotherapy is relatively safe and manageable for the treatment of oligometastatic prostate cancer (Table 7).

**Table 7:** Evaluation of radiotherapy-related complications of 66 patients undergoing radiotherapy.

Characteristics	RT (Primary lesion) (*n*=36)	RT (Metastatic lesion) (*n*=30)	*P*
CTCAE grade			0.5708
I	5	2	
II	1	1	
III	0	0	
IV	0	0	
V	0	0	
RTOG grade			0.4320
0	30	27	
1	6	3	
2	0	0	
3	0	0	
4	0	0	

CTCAE, Common Terminology Criteria for Adverse Events; RT, radiotherapy; RTOG, Radiation Therapy Oncology Group.

### Insights from retrospective cohort study

A retrospective cohort study comprising 102 patients diagnosed with oligometastatic prostate cancer was conducted to provide real-world insights into the comparative outcomes of CRP and RT. The key findings from this study were:

#### 5-year overall survival (OS)

**Figure 6:**
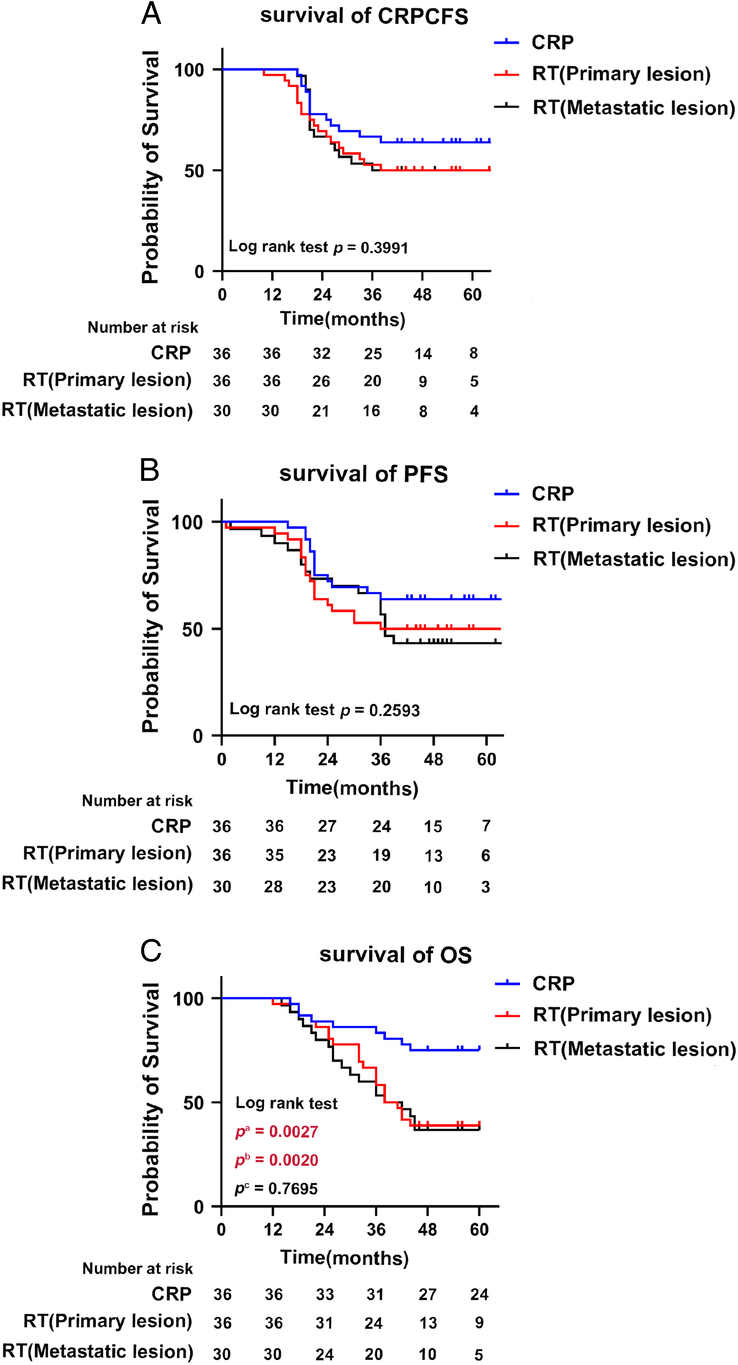
Kaplan–Meier estimates of primary and secondary endpoints for the OmPCa cohort underwent CRP: (A) Progression-free survival, (B) CRPC-free survival, and (C) Overall survival. CRP, cytoreductive radical prostatectomy; OmPCa, oligometastatic prostate cancer; RP, radiotherapy; CRPC, castration-resistant prostate cancer.^a^ Comparing RP with RT (Primary lesion) patients.^b^ Comparing RP with RT (Metastatic lesion) patients.^c^ Comparing RT (Primary lesion) with the RT (Metastatic lesion) cohort.

5-year OS was a significant endpoint assessed in the retrospective evaluation to evaluate the long-term influence of CRP versus RT on the survival rates. The results indicated that there exists an obvious difference in OS between the two groups (Log-rank, Chi-square, *P*=0.0034). The results of further subgroup analysis indicate that the OS in the CRP group is superior to that in the RT (Primary lesion) and RT (Metastatic lesion) groups, whereas no statistically significant difference is observed between the RT (Primary lesion) and RT (Metastatic lesion) groups. This finding suggests that patients who underwent cytoreductive surgery had a prolonged survival period than that of the other group (Table 8 and Fig. 6).

**Table 8:** Oncological outcomes in terms of PFS, CRPC-free survival, and 5-year OS.

	Patients, *n* (%)		
	CRP	RT (Primary lesion)	RT (Metastatic lesion)
PFS (SE) (median, IQR)	42.5 (21.8, 55.8)	39.0 (19.3, 52.0)	37.0 (19.8, 49.3)
HR (95% CI)^a^ ^b^ ^c^	0.62 (0.31, 1.26)	0.58 (0.28,1.19)	0.93 (0.48, 1.80)
*P* value^a^	0.1762		
*P* value^b^	0.1238		
*P* value^c^	0.8298		
CRPCFS (median, IQR)	43.0 (25.3, 56.0)	40.0 (21.25, 47.5)	39.0 (21.0, 46.0)
HR (95% CI)^a^ ^b^ ^c^	0.64 (0.31, 1.29)	0.68 (0.32, 1.43)	1.05 (0.53, 2.08)
*P* value^a^	0.2068		
*P* value^b^	0.2891		
*P* value^c^	0.8928		
5-year OS (median, IQR)	60.0 (45.0, 60.0)	32.0 (39.0, 59.0)	40.0 (25.8, 55.0)
HR (95% CI)^a^ ^b^ ^c^	0.33 (0.16, 0.67)	0.31 (0.15, 0.66)	0.91 (0.49, 1.69)
*P* value^a^	**0.0027**		
*P* value^b^	**0.0020**		
*P* value^c^	0.7695		

IQR, interquartile distance; CI, confidence interval; RP, radical prostatectomy; CRP, cytoreductive radical prostatectom; RT, radiotherapy; PSA, prostate-specific antigen; PFS, progression-free survival; CRPCFS, castration-resistant prostate cancer-free survival; OS, overall survival.

a Comparing RP with RT (Primary lesion) patients.

b Comparing RP with RT (Metastatic lesion) patients.

c Comparing RT (Primary lesion) with the RT (Metastatic lesion) cohort.

### Progression-free survival (PFS) and castration-resistant prostate cancer-free survival (CRPCFS)

Similar to the results of our previous meta-analysis, no significant differences were observed in progression-free survival (Log-rank, Chi-square, *P*=0.2593) or castration-resistant prostate cancer-free survival (Log-rank, Chi-square, *P*=0.3991) between the CRP and RT groups. Notably, there were no significant differences in PFS and CRPCFS between RT (Primary lesion) and RT (Metastatic lesion) groups (Table 8 and Fig. 6).

## Discussion

The management of oligometastatic prostate cancer remains a challenging and evolving field^23–26^, with the selection of appropriate treatment strategies significantly influencing patient outcomes and quality of life. In this study, we conducted a comprehensive meta-analysis alongside a real-world retrospective cohort investigation to compare the efficacy of cytoreductive surgery and radiotherapy for the treatment of oligometastatic prostate cancer. The combined approach of synthesizing existing evidence from the literature and analyzing outcomes within a well-defined patient cohort provides a comprehensive perspective on the relative benefits and limitations of these therapeutic modalities.

The meta-analysis incorporated data from five eligible studies, involving a substantial cohort of 1425 patients with oligometastatic prostate cancer. The results of the meta-analysis revealed that cytoreductive surgery demonstrated a significant improvement in OS when compared to radiotherapy [hazard ratio (HR): 0.80, 95% confidence interval [CI]: 0.77–0.82, *P*<0.001). Similarly, cytoreductive surgery exhibited superior cancer-specific survival (CSS) compared to radiotherapy (HR: 0.70, 95% CI: 0.59–0.81, *P*<0.001). These findings underscore the potential benefits of cytoreductive surgery in terms of extending survival outcomes, offering a valuable option for patients and clinicians.

In contrast, the analysis did not identify significant differences in progression-free survival (PFS) and castration-resistant prostate cancer-free survival (CRPCFS) between the cytoreductive surgery and radiotherapy groups (HR: 0.56, 95% CI: 0.17–1.06; HR: 0.67, 95% CI: 0.26–1.02, respectively). This suggests that while cytoreductive surgery may confer advantages in terms of OS and CSS, short-term disease control measures remain comparable between the two modalities. These results underscore the need to consider a range of factors when selecting treatment approaches, including patient preferences, tumor burden, and potential side effects.

The retrospective cohort study, conducted with institutional patient data, added further insights by providing real-world evidence of treatment outcomes. Involving 102 patients, the cohort study reaffirmed the improved OS in the cytoreductive surgery group when compared to the radiotherapy group. This finding was consistent with the meta-analysis results, thereby reinforcing the potential survival benefits associated with cytoreductive surgery for oligometastatic prostate cancer patients.

Furthermore, the cohort study’s breakdown of radiotherapy into primary lesion and metastatic lesion groups highlighted interesting distinctions. The superiority of cytoreductive surgery was evident in both overall survival and cancer-specific survival when compared to radiotherapy for the primary region. However, there was no significant difference in overall survival between radiotherapy for the primary region and radiotherapy for the metastatic region. This nuanced insight emphasizes the potential importance of targeting metastatic lesions directly through radiotherapy, potentially offering comparable survival outcomes for these patients.

The manageable adverse event rates observed in both cytoreductive surgery and radiotherapy modalities suggest that both treatment options are reasonably safe and well-tolerated. However, it is important to note that patient selection, comorbidities, and individual preferences should influence the choice of treatment to optimize outcomes while minimizing potential risks.

In conclusion, this study’s comprehensive analysis, combining a meta-analysis and a retrospective cohort study, contributes valuable insights into the comparative efficacy of cytoreductive surgery and radiotherapy for oligometastatic prostate cancer. The results highlight that while short-term survival measures are similar between the two modalities, cytoreductive surgery offers superior cancer-specific survival and overall survival outcomes. These findings provide clinicians with essential information for making informed treatment decisions tailored to individual patient circumstances. Nonetheless, it is crucial to acknowledge the study’s limitations, including potential selection biases in retrospective data, variations in treatment protocols, and the evolving definition of oligometastasis, particularly in light of advanced imaging technologies like PSMA-PET. This variability underscores the need for a standardized approach in defining oligometastasis to ensure consistency in future research. As a result, further prospective studies and randomized controlled trials are warranted to corroborate these findings and establish evidence-based therapeutic approaches for this distinct subset of patients.

## Conclusion

In conclusion, our comprehensive study addresses the clinical challenge presented by oligometastatic prostate cancer, offering insights into the comparative efficacy of cytoreductive surgery and radiotherapy. This investigation, comprising a meta-analysis and a retrospective cohort study, highlights the distinct benefits of cytoreductive surgery in the management of this condition. While short-term survival measures were comparable between the two groups, cytoreductive surgery exhibited superior cancer-specific survival and overall survival. The manageable adverse event rates associated with both modalities further enhance their viability as treatment options. These findings provide valuable guidance for clinicians managing oligometastatic prostate cancer patients, facilitating informed decision-making. However, to validate these results and guide personalized therapeutic approaches, future prospective studies and randomized controlled trials are imperative.

## Ethical approval

Not applicable.

## Consent

Not relevant.

## Sources of funding

There is no funding for this work.

## Author contribution

The authors of this paper participated in the study design. They also read, critiqued, and approved the manuscript revisions, as well as the final version of the manuscript. Also, all authors participated in a session to discuss the results and consider strategies for analysis and interpretation of the data before the final data analysis was performed and the manuscript written. All authors have the appropriate permissions and rights to the reported data.

## Conflicts of interest disclosure

No relevant conflicts of interest.

## Research registration unique identifying number (UIN)

1. Name of the registry: Assessment of Treatment Outcomes: Cytoreductive Surgery Compared to Radiotherapy in Oligometastatic Prostate Cancer – An In-Depth Quantitative Evaluation and Retrospective Cohort Analysis.
2. Unique identifying number or registration ID: CRD42023454107.
3. Hyperlink to your specific registration: https://www.crd.york.ac.uk/prospero/.

## Guarantor

Bisheng Cheng.

## Data availability statement

The datasets generated and/or analyzed during the current study are available from the corresponding author on reasonable request.

## Provenance and peer review

Not commissioned, externally peer-reviewed.

## References

[1] Sandhu S Moore CM Chiong E. Prostate cancer. Lancet 2021;398:1075–1090. 34370973 10.1016/S0140-6736(21)00950-8

[2] Lecouvet FE Oprea-Lager DE Liu Y. Use of modern imaging methods to facilitate trials of metastasis-directed therapy for oligometastatic disease in prostate cancer: a consensus recommendation from the EORTC Imaging Group. Lancet Oncol 2018;19:e534–e545. 30303127 10.1016/S1470-2045(18)30571-0

[3] Ost P Reynders D Decaestecker K. Surveillance or metastasis-directed therapy for oligometastatic prostate cancer recurrence: a prospective, randomized, multicenter phase II trial. J Clin Oncol 2018;36:446–453. 29240541 10.1200/JCO.2017.75.4853

[4] Wang Q Cheng B Singh S. A protein-encoding CCDC7 circular RNA inhibits the progression of prostate cancer by up-regulating FLRT3. NPJ Precis Oncol 2024;8:11. 38225404 10.1038/s41698-024-00503-2 PMC10789799

[5] Boeri L Sharma V Karnes RJ. Radiotherapy for newly diagnosed oligometastatic prostate cancer. Lancet 2018;392:2327–2328. 30355465 10.1016/S0140-6736(18)32598-4

[6] Connor MJ Winkler M Ahmed HU. Survival in oligometastatic prostate cancer-a new dawn or the Will Rogers phenomenon? JAMA Oncol 2020;6:185–186. 31804656 10.1001/jamaoncol.2019.4724

[7] Das A Shapiro DD Craig JK. Understanding and integrating cytoreductive nephrectomy with immune checkpoint inhibitors in the management of metastatic RCC. Nat Rev Urol 2023;20:654–668. 37400492 10.1038/s41585-023-00776-5

[8] Sidaway P. Cytoreductive surgery effective after relapse. Nat Rev Clin Oncol 2022;19:72. 10.1038/s41571-021-00589-8 34907328

[9] Romero D. Prospective evidence discourages secondary cytoreductive surgery. Nat Rev Clin Oncol 2020;17:68. 10.1038/s41571-019-0309-y 31804614

[10] Allen C Her S Jaffray DA. Radiotherapy for cancer: present and future. Adv Drug Deliv Rev 2017;109:1–2. 28189183 10.1016/j.addr.2017.01.004

[11] Overgaard J. Radiotherapy. Gazing at the crystal ball of European radiotherapy. Nat Rev Clin Oncol 2015;12:5–6. 25421280 10.1038/nrclinonc.2014.205

[12] Schaue D McBride WH. Opportunities and challenges of radiotherapy for treating cancer. Nat Rev Clin Oncol 2015;12:527–540. 26122185 10.1038/nrclinonc.2015.120 PMC8396062

[13] Page MJ McKenzie JE Bossuyt PM. The PRISMA 2020 statement: an updated guideline for reporting systematic reviews. Int J Surg 2021;88:105906. 33789826 10.1016/j.ijsu.2021.105906

[14] Mathew G Agha R Albrecht J. STROCSS 2021: Strengthening the reporting of cohort, cross-sectional and case–control studies in surgery. Int J Surg 2021;96:106165. 34774726 10.1016/j.ijsu.2021.106165

[15] Zhou L Zhou J Shuai H. Comparison of perioperative outcomes of selective arterial clipping guided by near-infrared fluorescence imaging using indocyanine green versus undergoing standard robotic-assisted partial nephrectomy: a systematic review and meta-analysis. Int J Surg 2024;110:1234–1244. 38000056 10.1097/JS9.0000000000000924 PMC10871632

[16] Tierney JF Stewart LA Ghersi D. Practical methods for incorporating summary time-to-event data into meta-analysis. Trials 2007;8:16. 17555582 10.1186/1745-6215-8-16 PMC1920534

[17] Yang Z Gong J Li J. The gap before real clinical application of imaging-based machine-learning and radiomic models for chemoradiation outcome prediction in esophageal cancer: a systematic review and meta-analysis. Int J Surg 2023, 109:2451–2466. 10.1097/JS9.0000000000000441 PMC10442126 37463039

[18] Culp SH Schellhammer PF Williams MB. Might men diagnosed with metastatic prostate cancer benefit from definitive treatment of the primary tumor? A SEER-based study. Eur Urol 2014;65:1058–1066. 24290503 10.1016/j.eururo.2013.11.012

[19] Patel DN Jha S Howard LE. Impact of prior local therapy on overall survival in men with metastatic castration-resistant prostate cancer: results from Shared Equal Access Regional Cancer Hospital. Int J Urol 2018;25:998–1004. 30253446 10.1111/iju.13806 PMC6279517

[20] Leyh-Bannurah SR Gazdovich S Budaus L. Local therapy improves survival in metastatic prostate cancer. Eur Urol 2017;72:118–124. 28385454 10.1016/j.eururo.2017.03.020

[21] Xue P Wu Z Wang K. Oncological outcome of combining cytoreductive prostatectomy and metastasis-directed radiotherapy in patients with prostate cancer and bone oligometastases: a retrospective cohort study. Cancer Manag Res 2020;12:8867–8873. 33061582 10.2147/CMAR.S270882 PMC7520542

[22] Lumen N De Bleser E Buelens S. The role of cytoreductive radical prostatectomy in the treatment of newly diagnosed low-volume metastatic prostate cancer. Results from the Local Treatment of Metastatic Prostate Cancer (LoMP) Registry. Eur Urol Open Sci 2021;29:68–76. 34337536 10.1016/j.euros.2021.05.006 PMC8317829

[23] Perez-Lopez R Tunariu N Padhani AR. Imaging diagnosis and follow-up of advanced prostate cancer: clinical perspectives and state of the art. Radiology 2019;292:273–286. 31237493 10.1148/radiol.2019181931

[24] DeSouza NM Liu Y Chiti A. Strategies and technical challenges for imaging oligometastatic disease: recommendations from the European Organisation for Research and Treatment of Cancer imaging group. Eur J Cancer 2018;91:153–163. 29331524 10.1016/j.ejca.2017.12.012

[25] Fossati N Giannarini G Joniau S. Newly diagnosed oligometastatic prostate cancer: current controversies and future developments. Eur Urol Oncol 2022;5:587–600. 33249083 10.1016/j.euo.2020.11.001

[26] Battaglia A De Meerleer G Tosco L. Novel insights into the management of oligometastatic prostate cancer: a comprehensive review. Eur Urol Oncol 2019;2:174–188. 31017094 10.1016/j.euo.2018.09.005

